# Antioxidant Effect of Propofol in Gliomas and Its Association With Divalent Metal Transporter 1

**DOI:** 10.3389/fonc.2020.590931

**Published:** 2020-11-24

**Authors:** Chenyi Yang, Zhengyuan Xia, Tang Li, Yimeng Chen, Mingshu Zhao, Yi Sun, Ji Ma, Yi Wu, Xinyue Wang, Peng Wang, Haiyun Wang

**Affiliations:** ^1^ Department of Anesthesiology, The Third Central Hospital of Tianjin, Nankai University Affinity the Third Central Hospital, The Third Central Clinical College of Tianjin Medical University, Tianjin, China; ^2^ Tianjin Key Laboratory of Extracorporeal Life Support for Critical Diseases, Artificial Cell Engineering Technology Research Center, Tianjin Institute of Hepatobiliary Disease, Tianjin, China; ^3^ Department of Anesthesiology, The University of Hong Kong, Hong Kong, China

**Keywords:** glioma, propofol, divalent metal transporter 1, α-amino-3-hydroxy-5-methylisoxazole-4-propionic acid receptor, oxidative stress

## Abstract

**Background:**

Oxidative stress enhances tumor invasion and metastasis in brain cancer. The activation of divalent metal transporter 1 (DMT1), which is regulated by glutamate receptors, can result in the increase of oxidative stress and risk of cancer development. Propofol, an anesthetic with antioxidant capacity, has been shown to decrease oxidative stress in several different types of cancer. However, the underlying mechanism remains unclear. Therefore, the present study aimed to elucidate the mechanism underlying the suppression of oxidative stress in glioma cells by propofol. It was hypothesized that propofol may inhibit oxidative stress in gliomas *via* suppressing Ca^2+^-permeable α-amino-3-hydroxyl-5-methylisoxazole-4-propionic acid (AMPA) receptor (CPAR)-DMT1 signaling.

**Methods:**

Male Wistar rats with C6 gliomas, which were established by intracranial injection of C6 glioma cells, were either treated with propofol or not for 6 h before being sacrificed. The levels of AMPA receptor subunit GluR2 and DMT1 protein expression were assessed using western blotting. The association between CPARs and DMT1 was confirmed *in vitro* using the AMPA receptor activator (R, S)-AMPA. Glutathione and reactive oxygen species assay kits were used to evaluate tumor oxidative stress. The effect of propofol on glioma proliferation was evaluated by determining tumor weight, cell cycles and a growth curve.

**Results:**

Propofol infusion at either 20 or 40 mg/kg^-1^/h^-1^ increased GluR2 levels and downregulated DMT1 expression as well as glutathione content markedly in the periphery compared with that in the glioma core. The *in vitro* results revealed that (R, S)-AMPA increased DMT1 expression and reactive oxygen species levels, which were partly reversed by propofol treatment.

**Conclusion:**

Propofol regulated DMT1 expression by modulating CPARs, resulting in the inhibition of tumor oxidative stress and glioma growth. The present study provides evidence for optimizing the selection of anesthetic drugs in perioperative management and prognosis of patients with glioma.

## Introduction

Of tumors in the central nervous system, >70% are gliomas, originating from astrocytes, oligodendrocytes and ependymal cells (1). The most common and fatal histological type is glioblastoma, which has a 5-year survival rate <5% in residents of England between 2007 and 2011 (2). An increasing number of studies have revealed that improper selection of anesthetics or anesthesia can adversely affect the prognosis of patients with glioma (3, 4). Therefore, optimizing the selection of anesthetic drugs in perioperative management has significant implications in the habilitation of patients with glioma. Propofol is widely used in clinical practice for intraoperative general anesthesia and postoperative sedation. Large amounts of evidence have confirmed that propofol inhibit the proliferation, invasion and migration, and facilitate apoptosis of tumor cells of various tissue origins, such as lung, gastrointestinal tract, pancreas, ovarian and cervical cancer (5–9). In addition, evidence has confirmed that propofol exhibits anti-oxidant capacity in various oxidative stress-induced diseases, such as Parkinson’s disease and myocardial, lung and hepatic ischemic/reperfusion injuries (10–14). However, the mechanism underlying the effect of propofol in tumors, particularly gliomas, remains largely unknown.

α-amino-3-hydroxy-5-methylisoxazole-4-propionic acid (AMPA) receptors combine with glutamate to mediate fast neurotransmission in excitatory synapses. AMPA receptors are also expressed in glial cells possessing the GluR2 subunit, which exhibits little Ca^2+^ permeability (15). However, AMPA receptors in glioma cells lacking the GluR2 subunit exhibit high Ca^2+^ permeability and are known as Ca^2+^-permeable AMPA receptors (CPARs) (16, 17). Researchers found that CPARs induce the proliferation and migration of human glioblastoma cells (18). In a neuron study, N-methyl-D-aspartic acid (NMDA)-type glutamate receptors, which are expressed at low levels in glioma cells, were found to regulate the expression of the iron transporter, divalent metal transporter 1 (DMT1) (19). DMT1 is the main protein that transports iron into cells during iron metabolism (20). The expression of DMT1 is closely associated with the intracellular iron content, whose excess levels contribute to oxidative stress and protein aggregation, resulting in neuronal death (21). Furthermore, DMT1 overexpression increases reactive oxygen species (ROS) levels (22). ROS generated by tumors are directly involved in malignant transformation, and oxidative stress plays a key role in tumor progression and angiogenesis (23).

An *in vitro* study illustrated that the functional NMDA receptors are lost in membranes of glioma cells, while CPAR (without GluR2 subunits) expression is increased and eventually becomes the major glutamate receptor that mediate Ca^2+^ influx (24). Thus, the present study hypothesized that CPARs may be involved in the upstream regulation of DMT1. It has previously been demonstrated that propofol downregulated CPAR expression in cerebral ischemia/reperfusion injury and conferred neuroprotective effects (25). The present study aimed to elucidate the mechanism underlying the suppression of oxidative stress in gliomas by propofol and to investigate whether propofol affects DMT1 expression by modifying CPARs and consequently influences the tumor redox status.

## Materials and Methods

### *In Vivo* Experiments

#### Animals

All animal experiments were approved by the Ethics Committee of Experimental Animals of Tianjin Medical University. A total of 96 male Wistar rats (weight, 250–300 g; age, 7–8 weeks) were provided by the China Academy of Military Medical Science and cared for according to the Guide for the Care and Use of Laboratory Animals (26). Before the experiments, the rats were anesthetized with intraperitoneal injections of 1% pentobarbital (40 mg/kg^-1^).

#### Cell Lines

The rat C6 glioma cells (C6 cells) were provided by Dr Zhuo Yang (Medical College, Nankai University, Tianjin, China). After resuscitation, the cells were incubated at 37°C in a 5% CO_2_ incubator and cultured in DMEM (high glucose; HyClone; Cytiva) containing 10% FBS (Biological Industries) and 1% antibiotic-antimitotic solution. The medium was changed daily using 75 cm^2^ culture flasks, passaged at least once until the logarithmic growth phase was reached, and prepared for further experiments.

For the *in vivo* tumor implantation, the passaged cells were washed with PBS and digested with 0.25% trypsin in DMEM for digestion. The supernatant was discarded after centrifugation (1000 rpm for 5 min at 22°C), and the cells were suspended in PBS to a final concentration of 10^6^ cells/10 μl.

#### Experimental Procedure

The rats were randomly divided into four groups: Sham (S; n=24 per group); glioma (G; n=24 per group); and propofol 20 and 40 mg/kg^-1^/h^-1^ (P1 and P2, respectively; n=24 per group) groups. After receiving anesthesia, the rats were placed in a stereotactic head frame (David Kopf Instruments), and craniotomy was performed. The glioma model was then established in all groups, except for the S group, using stereotactic implantation of C6 glioma cells into the right caudate nucleus (27, 28). On the 10th day after model establishment, propofol was infused intravenously at two doses at a rate of 20 and 40 mg/kg^-1^/h^-1^ in groups P1 and P2, respectively, using a syringe pump for 6 h. After 8 days, hematoxylin and eosin (H&E) staining (n=6/group) was performed and the glioma weight was measured (n=6/group). GluR2 and DMT1 expression was detected using western blotting (n=6/group) in the core and 2-mm diameter periphery of the tumors. Cerebrospinal fluid (CSF) was collected to determine the glutathione (GSH) content. Propofol was purchased from AstraZeneca Plc. The 60-day survival rate was investigated every day after propofol treatment (n=10/group). The tumor volume was monitored weekly with MRI for 60 days. Animals were euthanized with pentobarbital (150 mg/kg^-1^) before the study endpoint, which were: i) If the tumor size exceeded 13 mm in diameter; ii) the animal showed excessive weight loss (20% of body weight in a week), or iii) if the rat showed physiological signs of suffering (29).

#### Rat C6 Glioma Model

The procedure was performed 3 mm to the right of the midline and 1 mm anterior to the coronal suture. A total of 10 µl 10^6^ glioma cells was implanted stereotactically into the caudate nucleus 5 mm below the drill hole, which was subsequently sealed using bone wax, and the scalp was closed with an intermittent suture (27, 28).

#### Physiological Variables

For blood pressure measurement and sampling of arterial blood gases, the present study inserted polyethylene catheters into the right femoral artery. Body temperature was monitored with a rectal probe and was maintained at 37 ± 0.5°C using lamps. Physiological variables (mean arterial blood pressure, temperature and arterial blood gases) were measured before propofol treatment and 6 h after infusion.

#### Histological Observation

On the 18th day after the model was established, the rats were anesthetized and then euthanized with pentobarbital, followed by perfusion with formalin. The brains were subsequently removed and the weight of the fresh tumors was measured, followed by fixation in 10% formalin at a room temperature (RT) of 20 ± 2°C for 1 day. Coronal sections were cut at the level of the implanted tumor. The tissue blocks were paraffin-embedded and sectioned to obtain 7-μm thick slices, and then subjected to H&E staining. Glioma shape, tumor invasion range, necrosis and cellular arrangement were subsequently assessed.

#### Western Blotting

Tissues from the tumor core, 2-mm diameter tumor periphery of the tumor core and the ipsilateral hemisphere tissue from group S, were collected on day 18 after the model was established. Tissues were homogenized in lysis buffer (50 mmol/l^-1^ Tris-HCl pH 6.8, 150 mmol/l^-1^ NaCl, 5 mmol/l^-1^ EDTA, 0.5% sodium deoxycholate, 0.5% NP-40 and protease inhibitor cocktail) and centrifuged (12,000 rpm for 30 min at 4°C). For measuring GluR2 and DMT1 expression, a membrane protein extraction kit (APeXBIO Technology LLC) was used to isolate membrane proteins. Aliquots of the supernatant containing 50 μg protein were separated using 12% SDS-PAGE and immunoblotted onto polyvinylidene fluoride membranes. Then, the residual binding sites on the membrane were blocked by 5% skimmed milk powder for 1 h at RT. The membranes were incubated with primary antibodies (anti-GluR2 or anti-DMT1; both 1:1,000; both from Abcam; anti-GADPH, 1:7,000, ProteinTech Group, Inc.) for a night at 4°C. They were then incubated with the secondary antibodies (1:5,000; KPL, Inc.) for 1 h at RT. Each step was followed by washing with tris-buffered saline (TBS) plus Tween-20 (20 mM Tris, 150 mM NaCl and 0.1% Tween-20) 5 times for 5 min each time. Bands were visualized by exposing blots to x-ray film after incubation with freshly made chemiluminescent reagent (EMD Millipore), and then quantitatively analyzed using ImageJ software version 1.0 (National Institutes of Health) in a blinded fashion.

#### GSH Determination in CSF

Each rat was anesthetized and placed in the stereotaxic frame. The skin was prepared and an incision was made along the midline over the occipital crest. After separating the tissues, an angiocath catheter was used to puncture the occipital foramen magnum, and ~100 µl of CSF was slowly withdrawn over a 2 min period. To remove blood cells, the CSF samples were centrifuged at 300 g for 2 min. The GSH content was immediately determined using an enzymatic method with a commercial assay kit (Nanjing Jiancheng Bioengineering Institute) as described by Xia et al. (30).

### *In Vitro* Experiments

#### Experimental Process

C6 cell density was ~5×10^5^ cells/ml in the culture plate. For the *in vitro* study, the cells were divided into the following groups: Glioma (G); propofol 1.2 and 4.4 µg/ml^-1^ (P1 and P2, respectively); glioma treated with the AMPA receptor activator (R, S)-AMPA (100 µM, A + G); A + P1; and A + P2. The cells were passaged for 24 h until they reached 50%–70% confluency. They were then treated with (R, S)-AMPA and propofol for 6 h. The drug solutions were removed, and the cells were cultured for an additional 18 h to imitate the *in vivo* treatment. (R, S)-AMPA was purchased from Abcam. After the treatment, DMT1 expression and ROS levels were measured.

#### Immunofluorescence Staining and ROS Level Assay

The cells were grown on 6-well plates at a cell density of 30,000 cells/well and treated as previously described when cell confluence was up to 40% for live cell imaging. In the ROS assay, the cells were incubated in 25 µM of the reagent from the ROS analysis kit (Abcam) for 30 min at 37°C. The analysis of ROS levels was performed using a fluorescence microscope (magnification, ×200; Olympus Corporation). Images were captured from random 6 fields for each group, and data were analyzed using ImageJ software. The mean grey intensity value of each cell was then calculated manually.

#### Cell Cycle Analysis

Glioma cells were harvested, washed twice with PBS, fixed with pre-cooled 70% ethanol for a night at 4°C, and stored at -20°C for 12 h. The cells were then resuspended in PBS containing 25 mg/ml^-1^ propidium iodide, 0.1% Triton, and 10 mg/ml^-1^ RNase; incubated for 30 min at RT in the dark; and analyzed by flow cytometry. All samples were assessed with FACScan system (BD Biosciences, USA). Data were analyzed using FlowJo version 10.0.7r2 (BD Biosciences). Measurements were repeated six times.

#### Cell Proliferation Curve

The cells were digested and counted, 1x10^4^ cells of each group were plated in each of the wells in a 12-well dish with complete medium. The 6 wells of cells from each group were randomly selected, trypsinized and counted manually every 24 h for 6 days.

### Statistical Analysis

SPSS version 17.0 (SPSS Inc., USA) was used for the statistical analysis. Data are presented as the mean ± standard deviation. The survival rates were calculated using the Kaplan-Meier method and analyzed by log-rank test, with Bonferroni corrected P-values (P<0.0083) for multiple comparisons. Statistical analysis of C6 glioma cells growth curve were performed using two-way analysis of variance (ANOVA). Other results were analyzed using one-way ANOVA. After one-way or two-way ANOVA, Tukey’s multiple comparisons tests were applied. P<0.05 was considered to indicate a statistically significant difference.

## Results

### Physiological Variables During the Experiment

Physiological parameters, such as blood pressure, and temperature were closely monitored and controlled during the experimental period. There were no significant differences in these physiological parameters among rats in all groups during observation (before propofol treatment, and 6 h after infusion).

### Establishment of Rat C6 Glioma Model

**Figure 1:**
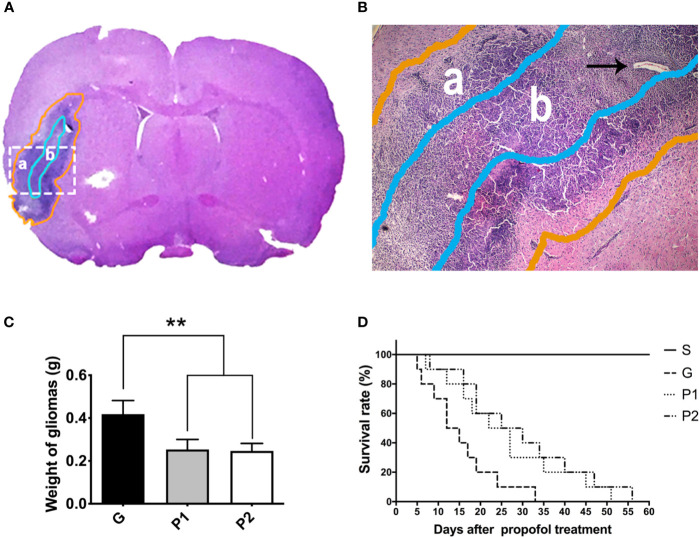
Shape of rat C6 glioma was revealed by whole-brain slice H&E staining, and 60-day survival curve was analyzed. **(A, B)** The shape of the glioma (a, gold circle) and tumor core (b, light blue circle) is shown using H&E staining of a tumor tissue coronal slice. Glioma cells and vascular arrangement (arrowhead) observed using optical microscopy (magnification, ×40). **(C)** On day 18 after establishing the C6 glioma model, the gliomas were removed from the rats and the weight of the fresh tumors was measured. Data are presented as the mean ± standard deviation (n=6/group). **(D)** The 60-day survival rate was investigated every day after propofol treatment (n=10/group). ^**^P < 0.01. Groups: G, glioma; P1 and P2, propofol 20, and 40 mg/kg^-1^/h^-1^, respectively. H&E, hematoxylin and eosin.

H&E staining revealed gliomas with irregular shapes and necrosis in the tumor core (**Figure 1A**). The necrotic regions were centrally coalesced, indicating a successfully established glioma model. Around the necrotic core region, typical palisading glioma cells were arranged in the peripheral part of the tumor. The tumor cells invaded the blood vessels (**Figure 1B**, arrowhead) up to the cerebral cortex, confirming that the rat C6 glioma model was successfully established on the right side of the caudate nucleus of the Wistar rats.

The glioma weight was analysed (**Figure 1C**). Compared with group G, glioma weight was lower in both group P1 (G vs. P1: 0.42 ± 0.06 vs. 0.25 ± 0.05; P<0.001) and group P2 (G vs. P2: 0.42 ± 0.06 vs. 0.25 ± 0.04; P<0.001), indicating that glioma growth was attenuated by propofol infusion. No differences between groups P1 and P2 were observed (P>0.05).

The survival rates were calculated using the Kaplan-Meier method and analyzed by the log-rank test. All the rats in group S survived during the investigation. Compared with group G, the survival time was extended in groups P1 (P=0.0092) and P2 (P=0.0356), suggesting that propofol infusion prolonged the survival time of C6 glioma rats, even though they were not significant after Bonferroni correction. Survival rates seemed higher in group P2 than in group P1, no statistical difference was observed between them (P>0.0083). These results suggest that propofol treatment can slightly delay the tumor progression of glioma rats (**Figure 1D**).

### Propofol Increased GluR2 Expression But Decreased DMT1 Expression and GSH Content *In Vivo*

**Figure 2:**
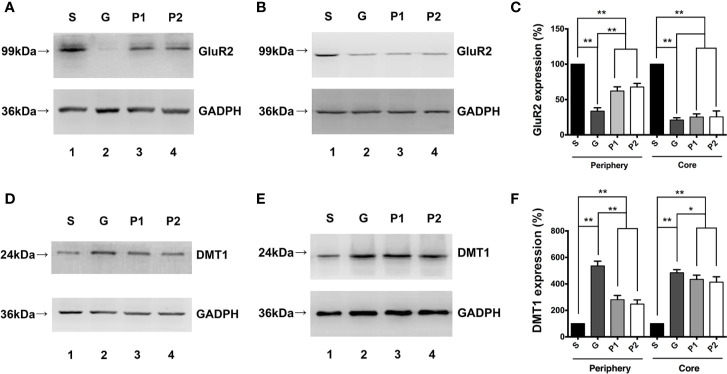
Effects of propofol infusion on GluR2 and DMT1 in two areas 18 days after glioma model establishment. **(A)** Western blotting result showed expression of GluR2 in the 2-mm diameter periphery of the gliomas. **(B)** Expression of GluR2 in the core of the gliomas. **(C)** Quantification of GluR2 expression and comparison within periphery and core groups. **(D)** DMT1 expression in the 2-mm diameter periphery of gliomas. **(E)** DMT1 expression in the core of the gliomas. **(F)** Quantification of DMT1 expression and comparison within the periphery and core groups. Data are presented as the mean ± SD (n=6/group). ^*^P < 0.05 and ^**^P < 0.01. Groups: S, sham; G, glioma; P1 and P2, propofol 20, and 40 mg/kg^-1^/h^-1^, respectively. DMT1, divalent metal transporter 1.

Western blotting revealed that GluR2 was expressed in both the 2-mm diameter periphery (**Figure 2A**) and tumor cores (**Figure 2B**) of the glioma, which was significantly decreased by tumor growth, compared with the expression levels observed in group S (S vs. G: 100 ± 0 vs. 33.56 ± 1.96; P<0.001, and S vs. P1: 100 ± 0 vs. 62.06 ± 2.45; P<0.001, and S vs. P2: 100 ± 0 vs. 67.86 ± 2.03; P<0.001, in the periphery; S vs. G: 100 ± 0 vs. 21.00 ± 1.31; P<0.001, and S vs. P1: 100 ± 0 vs. 25.26 ± 1.84; P<0.001, and S vs. P2: 100 ± 0 vs. 25.50 ± 3.43; P<0.001, in the core; **Figure 2C**). Propofol administration sharply increased the expression of GluR2 in the periphery (P<0.01). However, no statistical differences were observed between groups G and P1 or groups G and P2 in terms of GluR2 expression in the glioma core (**Figure 2C**). In addition, no significant difference existed between groups P1 and P2 in both areas. This finding suggests that propofol showed a greater effect in the tumor periphery than in the tumor core that was filled with glioma cells.

DMT1 expression results in both the 2-mm diameter periphery (**Figure 2D**) and tumor core (**Figure 2E**) appeared to be opposite to that of GluR2 expression. DMT1 expression was higher in group G than in group S (S vs. G: 100 ± 0 vs. 535.50 ± 14.69; P<0.001, in the periphery; **Figure 2F**). Propofol reversed this change effectively (G vs. P1: 535.50 ± 14.69 vs. 280.61 ± 13.18; P<0.001, and G vs. P2: 535.50 ± 14.69 vs. 247.13 ± 13.02; P<0.001, in the periphery; **Figure 2F**), but the levels did not reach those in the periphery of group S. In summary, DMT1 was inversely proportional to GluR2. Considering that gliomas mostly express CPARs with no GluR2 subunit on the membrane, it can be suggested that CPARs may be associated with DMT1 expression.

**Figure 3:**
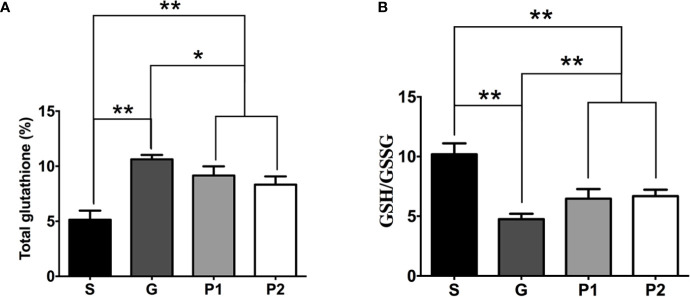
On day 18 after C6 glioma model establishment, CSF was withdrawn from rats, and the total GSH content and GSH/GSSG ratio was determined. **(A)** Quantification of total GSH content in CSF (n=6/group). **(B)** The analysis of GSH/GSSG ratio (n=6/group). Data are presented as means ± SD. ^*^P < 0.05 and ^**^P < 0.01. Groups: S, sham; G, glioma; P1 and P2, propofol 20, and 40 mg/kg^-1^/h^-1^, respectively. CSF, cerebrospinal fluid; GSH, glutathione; GSSG, glutathione disulfide.

The total GSH content significantly increased in groups G, P1, and P2 compared with group S, which may reflect increased oxidative stress in the tumors (S vs. G: 5.135 ± 0.34 vs. 10.62 ± 0.16; P<0.001, S vs. P1: 5.135 ± 0.34 vs. 9.16 ± 0.34; P<0.001, and S vs. P2: 5.135 ± 0.34 vs. 8.32 ± 0.30; P<0.0011; **Figure 3A**). Furthermore, the GSH/glutathione disulfide ratio increased following propofol infusion (G vs. P1: 4.75 ± 0.19 vs. 6.47 ± 0.33; P<0.01, G vs. P2: 4.75 ± 0.19 vs. 6.68 ± 0.22; P<0.001; **Figure 3B**), which reflects the antioxidant effects of propofol.

### CPARs Promotes Oxidative Stress and Glioma Cell Proliferation by Upregulating DMT1 *In Vitro*

**Figure 4:**
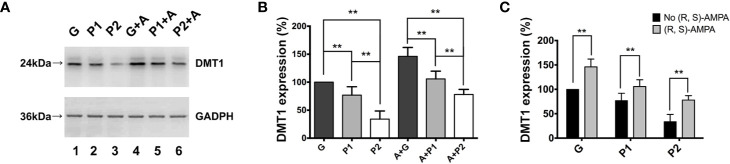
(R, S)-AMPA was incubated with tumor cells *in vitro* to confirm the relationship between Ca^2+^-permeable AMPA receptors and DMT1. **(A)** Western blotting result showed expression of DMT1 in glioma cells *in vitro*. **(B, C)** Quantification of DMT1 expression. Data are presented as mean ± SD (n = 6/group). ^**^P < 0.01. Groups: S, sham; G, glioma; P1 and P2, propofol 1.2, and 4.4 µg/ml^-1^, respectively; A, (R, S)-AMPA. AMPA, α-amino-3-hydroxy-5-methyl-4-isoxazolepropionic acid; DMT1, divalent metal transporter 1.

The present study used (R, S)-AMPA to confirm the association between CPARs and DMT1. The *in vitro* experiments demonstrated changes in DMT1 expression in all groups (**Figure 4A**). The expression tendencies of cells in groups G, P1, and P2 both with and without (R, S)-AMPA were similar to those observed *in vivo*. However, DMT1 expression was much higher in group P1 (A + P1) than in group P2 (A + P2), with significant differences (P1 vs. P2: 76.87 ± 6.16 vs. 33.88 ± 5.97; P<0.001, and A + P1 vs. A + P2: 105.90 ± 5.64 vs. 78.02 ± 3.69; P<0.01; **Figure 4B**). After culturing with (R, S)-AMPA, the DMT1 levels of the cells markedly increased and differed between groups G and (R, S)-AMPA (A) + G, as well as between P1 and A + P1 and between P2 and A + P2 (G vs. A + G: 100 ± 0 vs. 146.11 ± 6.51; P<0.001, P1 vs. A + P1: 76.87 ± 6.16 vs. 105.90 ± 5.65; P<0.01; and P2 vs. A + P2: 33.88 ± 5.9 vs. 78.02 ± 3.69; P<0.001). Propofol reversed these increases (all P<0.01). However, the DMT1 levels did not reach the levels detected before CPAR activation (**Figure 4C**).

**Figure 5:**
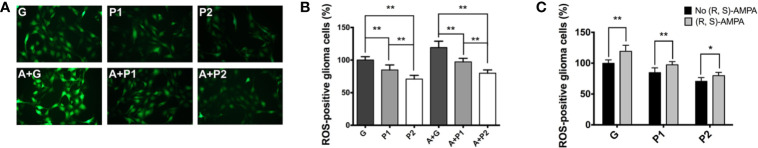
Oxidative stress status was investigated using ROS assay. **(A)** Fluorescence image of cellular ROS productive levels (magnification, ×200). **(B, C)** Quantification of ROS levels in the gliomas. Data are presented as the mean ± SD (n=6/group). ^*^P < 0.05 and ^**^P < 0.01. Groups: S, sham; G, glioma; P1 and P2, propofol 1.2, and 4.4 µg/ml^-1^, respectively; A, (R, S)- AMPA. ROS, reactive oxygen species; AMPA, α-amino-3-hydroxyl-5-methyl-4-isoxazolepropionic acid.

The ROS levels were directly proportional to DMT1 expression, as presented in **Figure 5A**. The administration of (R, S)-AMPA exacerbated oxidative stress in glioma cells (**Figure 5C**), which may increase the demand for reducing agents and accelerate tumor metabolism and deterioration. Consistently, propofol inhibited ROS production (G vs. P1: 100 ± 2.13 vs. 84.83 ± 3.14; P<0.01, and G vs. P2: 100 ± 2.13 vs. 70.83 ± 2.37; P<0.01; A + G vs. A + P1: 119.28 ± 4.01 vs. 97.33 ± 2.17; P<0.001, and A + G vs. A + P2: 119.28 ± 4.01 vs. 79.83 ± 2.09; P<0.001; **Figure 5B**), thereby preventing oxidative stress in gliomas.

**Figure 6:**
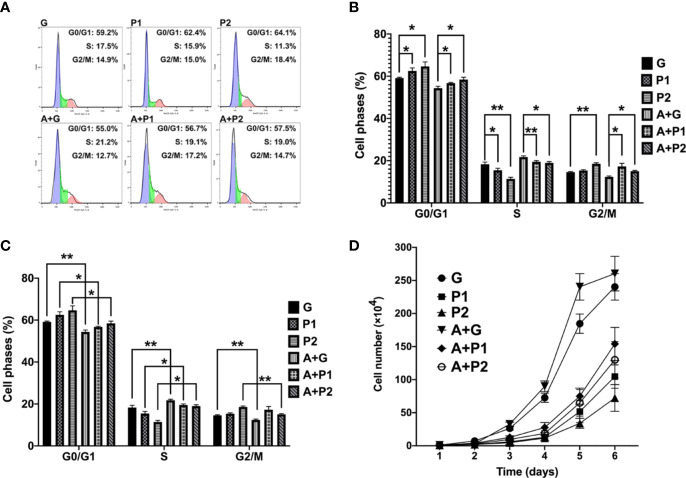
It was revealed that propofol suppressed proliferation and cell cycle progression of glioma cells. **(A–C)** Cell cycle analysis of C6 glioma cells transduced with propofol or (R, S)-AMPA and analyzed by flow cytometry. **(D)** For the cell proliferation assay, 6 wells of cells from each group were randomly selected, digested and counted every 24 h for 6 days. Data are presented as the mean ± SD (n=6/group). ^*^P < 0.05 and ^**^P < 0.01. Groups: S, sham; G, glioma; P1 and P2, propofol 1.2, and 4.4 µg/ml^-1^, respectively; A, (R, S)- AMPA.

A decrease in cell proliferation is often accompanied by changes in cell cycle progression. Therefore, the present study performed a cell cycle analysis and showed the administration of (R, S)-AMPA increased tumor cell proliferation through escalating the numbers of cells in S and G_2_/M phases, and reducing the numbers of cells in G_0_/G_1_ phase (**Figure 6C**). Cell cycle analysis also identified a marked increase induced by propofol in the numbers of cells arrested in the G_0_/G_1_ phase (G vs. P1: 59.17 ± 0.35 vs. 62.50 ± 1.45; P<0.05, and G vs. P2: 59.17 ± 0.35 vs. 64.63 ± 2.15; P<0.05; A + G vs. A + P1: 54.33 ± 0.91 vs. 56.73 ± 0.35; P<0.05, and A + G vs. A + P2: 54.33 ± 0.91 vs. 58.43 ± 1.07; P<0.05), while lowering the numbers of cells in S (G vs. P1: 18.30 ± 1.06 vs. 15.43 ± 0.99; P<0.05, and G vs. P2: 18.3 ± 1.06 vs. 11.37 ± 0.80; P<0.01; A + G vs. A + P1: 21.73 ± 0.55 vs. 19.50 ± 0.53; P<0.01, and A + G vs. A + P2: 21.73 ± 0.55 vs. 18.97 ± 0.65; P<0.05) and G_2_/M phases (G vs. P1: 14.57 ± 0.31 vs. 15.33 ± 0.42; P>0.05, and G vs. P2: 14.57 ± 0.31 vs. 18.57 ± 0.47; P<0.01; A + G vs. A + P1: 12.37 ± 0.42 vs. 17.27 ± 1.50; P<0.05, and A + G vs. A + P2: 12.37 ± 0.42 vs. 15.07 ± 0.32; P<0.05), as compared with the G or A+G group (**Figures 6A, B** **)**. The present study then performed a C6 glioma cells proliferation assay. Proliferation curves revealed that propofol significantly decreased the proliferation rate (G vs. P1: P<0.0001, and G vs. P2: P<0.0001; A + G vs. A + P1: P=0.0003, and A + G vs. A + P2: P=0.0001; **Figure 6D**). These data demonstrated that propofol maintained or prolonged C6 glioma cells in the G_1_-phase and, as a consequence, decreased tumor cell proliferation. The effect of propofol on glioma cell cycles and proliferation was consistent with that on glioma weight. Altogether, the *in vitro* results in the present study supported the *in vivo* results, which suggested that propofol serves as a tumor suppressor in glioma.

## Discussion

The present study aimed to elucidate the mechanism underlying the suppression of oxidative stress in gliomas by propofol and revealed that the effect of propofol on glioma cell cycles and proliferation was consistent with its effects on the 60-day survival rate. Taken together, the *in vitro* results of the present study supported the *in vivo* results, indicating that propofol may serve as a tumor suppressor in gliomas. Furthermore, propofol increased GluR2 and decreased glioma weight as well as DMT1 expression, with the effects being more apparent in the tumor periphery than in the core. Propofol decreased the GSH content in CSF, which may be a consequence of inhibition of oxidative stress in gliomas by propofol.

DMT1 is an iron importer protein responsible for ferrous iron influx. It is regulated by NMDA receptors in neurons (19). However, NMDA receptor expression in glioma cells is low. CPARs are vital glutamate receptors that are Ca^2+^-permeable. Thus, the present study surmised that CPARs may be involved in the upstream regulation of DMT1. To confirm this hypothesis, the present study performed *in vitro* experiments using (R, S)-AMPA, a CPAR activator. The *in vitro* findings supported the hypothesis, as treatment with (R, S)-AMPA increased DMT1 expression following CPAR expression upregulation, suggesting that CPARs may be involved in the upstream regulation of DMT1 in glioma cells. Yet propofol decreased DMT1 expression and tumor cell proliferation, which suggested that propofol partly reversed the effects of (R, S)-AMPA. In addition, immunofluorescence staining results showed that ROS production was significantly increased in group G, while propofol lowered the tumor ROS level. In a previous study, pancreatic β-cell iron depletion with two iron chelators and DMT1 deletion by both siRNA and transgenic approaches decreased IL-1β-induced ROS production and β-cell apoptosis *in vitro*, demonstrating the association between DMT1 and ROS (31). In line with these findings, the present study further demonstrated that propofol decreased ROS in tumors *via* regulation of DMT1. Previous published works demonstrating the antitumor effect of propofol in glioma were mainly based on *in vitro* studies, illustrated that propofol suppresses proliferation and invasion of glioma cells by upregulating microRNA-218 expression, inhibiting Wnt signaling or blocking the PI3K/Akt pathway through miR-206/ROCK1 axis (32–34). The present study revealed propofol anti-tumor effect from the perspective of oxidative stress inhibition in glioma cells through regulating DMT1 expression by modifying CPARs. Despite the lack of intracellular Ca^2+^ and Fe^2+^ tests, which is a limitation of the present study, to the best of our knowledge, the present study is the first to show that propofol decreases oxidative stress *via* a novel mechanism of DMT1 regulation.

**Figure 7:**
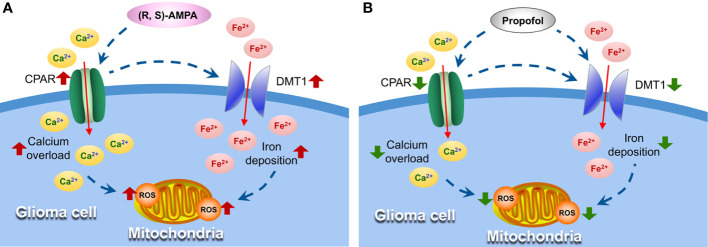
Propofol suppresses oxidative stress in glioma cells by inhibiting DMT1 and CPAR expression. **(A)** AMPA receptor activator (R, S)-AMPA induces CPARs upregulation and Ca^2+^ influx, which can cause the excessive expression of DMT1, followed by increased ROS formation in mitochondria. **(B)** Propofol inhibits CPAR and DMT1 expression and eventually suppresses oxidative stress and tumor growth. DMT1, divalent metal transporter 1; AMPA, α-amino-3-hydroxyl-5-methyl-4-isoxazolepropionic acid; CPAR, Ca^2+^-permeable AMPA receptors; ROS, reactive oxygen species.

The GSH content in the CSF and the cellular ROS production were significantly increased in group G, while propofol lowered tumor GSH and ROS levels. The expression and activity of glutamate transporters [excitatory amino acid transporters (EAAT) 1 and EAAT2] have been reported to be significantly decreased in both glioma cell lines and fresh glioma tissues (the ability to transport glutamate was only 1/100 in the physiological state) (35). Furthermore, the cystine-glutamate antiporter ($x_{c}^{-}$ system) activity increased (glutamate released was three times higher than the normal levels) (35). System $x_{c}^{-}$ releases large amounts of glutamate, which is important for promoting glioma growth and for maintaining its invasiveness (36). Hypoxia usually occurs in gliomas due to its excessive growth and metabolism, while energy is mainly generated by anaerobic glycolysis, leading to intense generation of ROS. The elimination of intracellular ROS primarily relies on GSH, which is synthesized from cysteine. The accumulation of cellular ROS would thus enhance system $x_{c}^{-}$ activity to meet the requirement for GSH (37). It has been reported that extracellular glutamate increased with $x_{c}^{-}$ upregulation, which in turn induces Ca^2+^ influx through CPARs, and the latter can then induce calcium overload in the mitochondria and produce abundant ROS (38, 39). Sato et al. (40) recently demonstrated that the ferroptosis inducer erastin irreversibly inhibits system $x_{c}^{-}$ leading to cysteine starvation, glutathione depletion, and consequently ferroptotic cell death, and efficiently kills human tumor cells without killing their isogenic normal cell counterparts. Ferroptosis refers to a novel iron-dependent form of regulated necrotic cell death (41). Hence, iron metabolism and system $x_{c}^{-}$ have recently emerged as potential targets in the context of cancer therapy. It was previously observed that propofol decreased CPAR expression, which inhibits calcium transport to reduce ischemia/reperfusion injury (25). It was recently revealed that propofol could inhibit CPARs and decrease viability, invasiveness and migration of C6 glioma cells (42). The results of the present study show that propofol inhibits oxidative stress and DMT1 expression and eventually downregulates GSH and ROS production, which disrupts the energy supply required for tumor growth and metabolism (**Figure 7**).

In the *in vivo* experiments in the present study, propofol doses of 20 and 40 mg/kg^-1^/h^-1^, which corresponded to 1.2 and 4.4 µg/ml^-1^ of propofol for incubating cells *in vitro*, were selected and determined based on the following findings. In previous research on ischemia/reperfusion injury, compared with propofol infusion rate of 35 mg/kg^-1^/h^-1^, propofol at doses of 10 and 20 mg/kg^-1^/h^-1^ were found to confer improved protective effects against brain functional injury and ischemia/reperfusion injury, which had no statistic difference between them (25). In addition, using 10 mg/kg^-1^/h^-1^ propofol alone could barely maintain general anesthesia, let alone provide sedation to rats. Therefore, the present study selected the sub-anesthetic dose of 20 mg/kg^-1^/h^-1^ to continue the further studies. Logginidou et al. (43) reported that the corresponding estimated plasma concentration of propofol was 1.2 µg/ml^-1^ when the infusion rate was 20 mg/kg^-1^/h^-1^. Furthermore, the steady-state mean arterial blood propofol concentration for general anesthesia was 4.4 µg/ml^-1^, when the infusion speed was 40 mg/kg^-1^/h^-1^ *in vivo*, which was selected for comparison (43, 44). Nevertheless, propofol at doses of 36 and 72 mg/kg^-1^/h^-1^ exacerbated brain injury, hindered post-traumatic neurogenesis, and increased the 28-day mortality after experimentally-induced trauma, which demonstrated that propofol brain protective effect was not dose-dependent (45). The present study revealed that propofol infusion rates of 20 and 40 mg/kg^-1^/h^-1^ caused different effects in gliomas, but led to no statistical differences *in vivo*. This observation could be explained by the interference of other factors in rats; however, the cell culture environment was less complex. Glioma cells were much more vulnerable to propofol than rats, and the increase in propofol dose *in vitro* may produce significant differences compared with a sub-dose. In addition, the follow-up study will continuously observe the long-term effects of propofol on tumors, since the effect of ROS on tumors may be neither constant nor unique (46).

## Conclusion

In the present study, it was revealed that propofol regulates DMT1 expression by modifying CPARs, thereby inhibiting tumor oxidative stress and tumor growth. The findings of the present study suggested that perioperative propofol application may help achieve good quality of glioma prognosis, although clinical trials are needed to test this intriguing hypothesis, as various factors affected the condition of patients with glioma. In clinical studies, it is difficult to conclude whether any anesthetic at any certain dose has marked antitumor effects. However, from the perspective of anesthesiologists, it is necessary to minimize the risk of long-term metastasis and recurrence, and provide safe, comfortable and high-quality anesthesia management in patients with glioma.

## Data Availability Statement

The raw data supporting the conclusions of this article will be made available by the authors, without undue reservation.

## Ethics Statement

The animal study was reviewed and approved by the Committee of Experimental Animals of Tianjin Medical University.

## Author Contributions

HW, ZX, and CY designed the present study. CY, TL, YC, XW, and PW performed the experiments. CY, MZ, YS, JM, and YW analyzed the data. CY, HW, and ZX wrote the article. All authors contributed to the article and approved the submitted version.

## Funding

The present study was supported by grants from the National Natural Science Foundation of China (Grant No. 82071220), the Natural Science Foundation of Tianjin (Grant No. 20JCYBJC01290), the Tianjin Major Support Program of Science and Technology (Grant No. 18YFZCSY00530), the Tianjin Research Program of Application Foundation and Advanced Technology (Grant No. 15JCYBJC25600), and the Young Scholar Research Grant of Chinese Anesthesiologist Association (Grant No. 21800004).

## Conflict of Interest

The authors declare that the research was conducted in the absence of any commercial or financial relationships that could be construed as a potential conflict of interest.

## Abbreviations

AMPA, α-amino-3-hydroxy-5-methylisoxazole-4-propionic acid; CPARs, Ca^2+^-permeable AMPA receptors; CSF, cerebrospinal fluid; DMT1, divalent metal transporter 1; EAAT, excitatory amino acid transporters; FBS, fetal bovine serum; GSH, glutathione; ICUs, intensive care units; IRE, iron-response element; NMDA, N-methyl-D-aspartic acid receptor; ROS, reactive oxygen species.
